# Supervised Hyperspectral Band Selection Using Texture Features for Classification of Citrus Leaf Diseases with YOLOv8 †

**DOI:** 10.3390/s25041034

**Published:** 2025-02-09

**Authors:** Quentin Frederick, Thomas Burks, Jonathan Adam Watson, Pappu Kumar Yadav, Jianwei Qin, Moon Kim, Megan M. Dewdney

**Affiliations:** 1 Department of Agricultural and Biological Engineering, University of Florida, P.O. Box 110570, Gainesville, FL 32611-0570, USA; qfrederick@ufl.edu (Q.F.); jaw7385@ufl.edu (J.A.W.); 2 Department of Agricultural and Biosystems Engineering, South Dakota State University, P.O. Box 2100, Brookings, SD 57007, USA; pappu.yadav@sdstate.edu; 3 USDA/ARS Environmental Microbial and Food Safety Laboratory (EMFSL), Beltsville Agricultural Research Center, Beltsville, MD 20705, USA; jianwei.qin@usda.gov (J.Q.); moon.kim@usda.gov (M.K.); 4 Citrus Research and Education Center, 700 Experiment Station Rd., Lake Alfred, FL 33850, USA; mmdewdney@ufl.edu

**Keywords:** hyperspectral imagery (HSI), citrus inspection, leaf inspection, band selection, feature extraction, YOLOv8, huanglongbing, citrus canker

## Abstract

Citrus greening disease (HLB) and citrus canker cause financial losses in Florida citrus groves via smaller fruits, blemishes, premature fruit drop, and/or eventual tree death. Management of these two diseases requires early detection and distinction from other leaf defects and infections. Automated leaf inspection with hyperspectral imagery (HSI) is tested in this study. Citrus leaves bearing visible symptoms of HLB, canker, scab, melanose, greasy spot, zinc deficiency, and a control class were collected, and images were taken with a line-scan HSI camera. YOLOv8 was trained to classify multispectral images from this image dataset, created by selecting bands with a novel variance-based method. The ‘small’ network using an intensity-based band combination yielded an overall weighted F1 score of 0.8959, classifying HLB and canker with F1 scores of 0.788 and 0.941, respectively. The network size appeared to exert greater influence on performance than the HSI bands selected. These findings suggest that YOLOv8 relies more heavily on intensity differences than on the texture properties of citrus leaves and is less sensitive to the choice of wavelengths than traditional machine vision classifiers.

## 1. Introduction

The citrus industry contributes significantly to the economy of the state of Florida, which is currently the top domestic supplier for the U.S. orange juice market [1]. Although a small fraction of the peak production of 300 million boxes per year was recorded in the late 1990s [2], in the 2023–2024 crop year, Florida produced 20.2 million boxes of citrus [3]. Among fruit production, juice production, and marketing, a sum of approximately USD 6.9 billion was contributed to Florida’s economy over the 2020–2021 season [4].

This decline can be attributed to a few factors, including hurricane damage, urban growth, a labor shortage, and especially to the recent rise of a few invasive pathogens. A statewide reduction of production value of approximately USD 1 billion over a recent 10-year period has been measured, along with commensurate losses in overall production and bearing acreage [4]. Since being found in Florida in 2005 [5], Huanglongbing (HLB; citrus greening) has become one of the most significant threats to the state’s citrus industry. Transmitted by the Asian citrus psyllid (*Diaphorina citri*), HLB is a bacterial infection of *Candidatus Liberibacter asiaticus*. An infected tree will display symptoms including mottling on leaves, twig dieback, premature fruit drop, and gradual tree decline. Also, fruit from infected trees is often of compromised quality [6]. Injection of oxytetracycline into tree trunks can suppress the bacterial population [7] and reduce the effect of HLB on fruit [8], but it does not cure existing infections. Preventing infection by placing citrus groves underneath mesh screens, known as citrus under protective screen (CUPS), is also being attempted [9], but the long-term feasibility of this approach is not yet known. Currently, the recommended defenses against HLB are focused on prevention, including removal of infected trees and psyllid control [6,10].

Citrus canker is a bacterial infection caused by some species of the *Xanthomonas* genus and has been subject to eradication efforts multiple times over the last century. Canker is transmitted by wind-blown rain, and infected fruit and leaves bear raised lesions surrounded by a yellow halo. Canker also causes fruit drop and twig dieback [11]. Florida is a quarantined area for canker, requiring meeting additional regulations for shipping fruit out of the state [12]. Fruit can be protected from infection by copper sprays, but this method is much less effective on leaves since they grow faster [13].

Distinguishing symptoms of HLB and canker from those of more easily treatable conditions has been recognized as a difficulty in grove inspection [14]. Melanose, scab, and greasy spot are also found in Florida but can be controlled with pesticides and thus are not as costly as HLB and canker [15]. Accurate detection and mapping of citrus diseases enables and improves the effectiveness of other management tools. Effective disease management not only benefits growers but also preserves jobs in fruit production, juice processing, and marketing, contributing to the overall economic stability in Florida’s agricultural sector.

Previous citrus leaf disease inspection with conventional machine vision has taken advantage of both color and textural features. In 2006, Pydipati et al. [16] showed that higher contrast between the leaf surface and the lesions in RGB imagery facilitated detection of scab and melanose (with accuracies mostly above 95%) on the back side of grapefruit leaves. Early detection and accurate classification of diseases may prevent widespread infection, reducing the need for extensive pesticide use and tree removal.

Compared to RGB images, the higher spectral resolution and range of hyperspectral imagery (HSI) offer advantages for food inspection. Imaging dozens or hundreds of narrow bands instead of the three broad bands produced by RGB cameras can facilitate detection by accentuating defects. It has been shown that analysis of HSI benefits from consideration of both spatial and spectral features [17]. Nectarines [18], peaches [19], jujubes [20], pomegranates [21], and mangoes [22] have all been inspected with HSI. It has also been employed to inspect spinach leaves [23] and detect foreign objects in dried seaweed [24]. In addition to research efforts, commercial applications of HSI to food inspection have proven successful [25,26]. Applications will likely become more ubiquitous as hardware becomes more compact and inexpensive [27,28]. Cardinali et al. [29] found spectral differences between healthy and HLB-infected leaves, indicating that HSI can permit HLB detection.

For the purposes of both in-field and packinghouse disease inspection, these systems should operate in real time to acquire imagery and discriminate conditions of interest at real-time speeds. Consequently, hyperspectral data, although important in preliminary investigations for identifying wavelengths of interest, is neither computationally efficient nor cost-effective in practical applications. Therefore, it is important to collect hyperspectral data for analysis and detection system development, but with the intention of later transitioning to multispectral systems, which are typically limited to 3–5 bands. In addition to this pragmatic reason to select bands, adjacent bands are often redundant information because their bandpass center frequencies are adjacent [30].

Various mathematical methods, including manual techniques [31], orthogonal subspace projection [32], entropy distance [33], genetic algorithms [34], and sparse linear discriminant analysis (LDA) [35], have been applied to mathematically map an HSI spectrum to a smaller number of bands, but these methods require numerous original bands as inputs. Moreover, bands resulting from such mathematical operations are less interpretable than selected bands [36].

Frederick et al. [37] evaluated the HSI band selection method on a dataset of images of orange peels belonging to five classes. Both a supervised method (using class labels) and an unsupervised one (not requiring class labels) were tested. Both methods selected 1–5 bands, which were used to train a custom CNN. A model using SVM to classify five bands selected with principal component analysis (PCA) yielded a peak overall accuracy of 94.9%. However, with two bands or fewer, the supervised bands yielded better classification with either classifier.

Gray-level co-occurrence (GLCM) is a well-known, widely used texture feature extraction method [38] based on relative frequency distributions [39]. It has been applied to inspect crops and fresh produce, including bananas [40], pomegranates [41], and grape leaves [42]. Gómez-Flores et al. [43] used GLCM features for multiclass citrus leaf disease inspection, reaching 81% accuracy. Likewise, HSI bands from imagery of tomatoes [44] and orchard trees [45] have been selected with analysis of variance (ANOVA). By testing the separability of class means in each feature, ANOVA can be employed to ‘score’ each feature independently of all the others.

In recent years, deep learning (DL) has become a popular means of analyzing imagery across several disciplines. For remote sensing, the You Only Look Once (YOLO) series of networks offers the options of both spectral and spatial feature extraction [46]. The YOLO series of CNNs can both segment images for object detection and classify entire images. Danajayan et al. [47] trained YOLOv4 on a 2684-image dataset to detect anthracnose, bacterial brown spot, and melanose on citrus leaves. Asian citrus psyllids, the HLB vector, have been detected in outdoor images with variations of YOLOv5 [48]. Three variations of YOLOv5 were trained on RGB datasets of citrus leaves and fruit with both HLB and similar symptoms for HLB detection, recording a peak F1 score of 85% [49]. A comparison of YOLOv5, YOLOv7, and YOLOv8 for classification of diseases on leafy vegetables favored YOLOv8, which achieved 100% test accuracy [50]. Variations of the YOLOv8 architecture have also been shown to be effective at disease detection in the presence of multiple disease classes. Infections on maize and rice leaves have been detected with variations of the YOLOv8 architecture and loss function [51,52]. Nguyen et al. [53] segmented and classified diseased grapevines with a CNN feature extractor and HSI.

Despite only being available since 2023 [54], YOLOv8 has been applied to inspecting citrus leaves and fruit. Lu et al. [55] found that despite having fewer parameters than other tested models, YOLOv8 was the second-best performing citrus peel inspection model. Luo et al. [56] segmented and classified six types of surface defects on citrus leaves and peels with 93% accuracy and 93% mAP using a variation of YOLOv8 trained on a 3000-image RGB dataset of citrus leaves and fruits.

Both intensity- and texture-based features have contributed to plant inspection [57]. Despite CNNs’ proven performance with RGB imagery, little work has combined HSI with YOLOv8 for citrus diseases. Previous work has indicated that supervised band selection improved classification [58], but did not answer whether the major error modes were limitations of the dataset, the classifier, or the bands selected. Nor have previous studies considered spatial relationships when selecting bands for leaf inspection, despite CNNs’ suitability for leaf inspection with texture features [59]. This study demonstrates multiclass classification of diseased citrus leaves with multiple variants of the YOLOv8 architecture using hyperspectral imagery and tests the effect of involving texture in band selection.

The objectives of this study are as follows:

- Train YOLOv8 to classify citrus leaf images of seven disease classes (HLB, canker, zinc deficiency, scab, melanose, greasy spot, and control) composed of bands selected with three methods: ANOVA ranking of pixel values, ANOVA ranking of GLCM texture features, and random choice.
- Compare the overall and class-specific classification results of these trained models.
- Evaluate the sensitivity of the classification model to the choice of wavelengths selected and the size of the classifier network.

## 2. Materials and Methods

### 2.1. Data Collection

Approximately 750 citrus leaves were collected from groves near the Citrus Research and Education Center (CREC) (Lake Alfred, FL, USA). CREC personnel collected healthy leaves and leaves bearing symptoms of the following infections: HLB, citrus canker, melanose, greasy spot, zinc deficiency, and citrus scab. Leaves with symptoms of multiple infections were excluded. All classes used Valencia orange leaves, except for scab, which required leaves from ‘Furr’ mandarin trees. Both adaxial and abaxial sides of each leaf were imaged within 48 h of collection, with leaves refrigerated in the meantime. The HSI system permitted 4–16 leaves to be imaged simultaneously.

**Figure 1:**
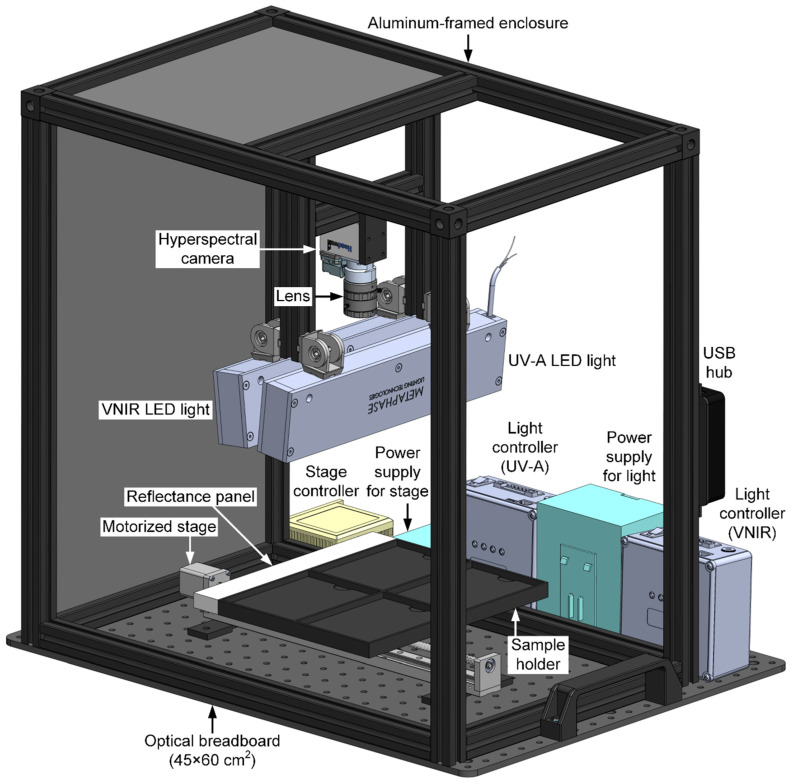
A CAD rendering displaying components of the portable HSI system developed at USDA ARS EMFSL used to image leaves for this study. For clarity, enclosure panels are omitted.

Developed recently at the USDA ARS EMFSL, the HSI system displayed in Figure 1 images 348 spectral bands in a wavelength range of 395–1005 nm. A 254 × 32 × 15 mm^3^ reflectance standard panel (Labsphere of North Sutton, NH, USA) is mounted alongside a black, thermoplastic 254 × 197 × 15 mm sample tray.

A miniature line-scan hyperspectral camera (Nano-Hyperspec VNIR, Headwall Photonics, Bolton, MA, USA) captures both reflectance and fluorescence signals in the VNIR range. This instrument integrates an imaging spectrograph and a CMOS focal plane array detector (12-bit and 1936 × 1216 pixels). A lens with a 5 mm focal length (Edmund Optics, Barrington, NJ, USA) attached to the camera provides a wide-angle view. Also, a long-pass gelatin filter (>400 nm, Kodak, Rochester, NY, USA) removes second-order effects from the UV-A excitation. When set at a lens-to-sample distance of 285 mm, the spatial resolution is 0.33 mm/pixel. During scanning, the sample holder (and the reflectance panel) is translated beneath the hyperspectral camera by a linear motorized stage (FUYU Technology of Chengdu, Sichuan, China). An 810 × 348 (spatial × spectral) pixel region of interest (ROI) is extracted from each frame, covering a spectral range of 395–1005 nm.

Two LED line lights (Metaphase Technologies, Bristol, PA, USA), angled at approximately 6° from the vertical position, illuminate the samples. For reflectance imaging, these provide both visible and near-infrared broadband light at seven wavelengths: 428, 650, 810, 850, 890, 910, and 940 nm. For fluorescence imaging, the line lights provide ultraviolet-A (UV-A) excitation light at 365 nm. At these eight wavelengths, intensities can be adjusted with two digital dimming controllers, each with three channels. Specifically, four channels are used to regulate the intensities at 365, 428, 650 nm, and a bundle of five NIR wavelengths (810, 850, 890, 910, and 940 nm).

The entire system is framed with aluminum extrusions on an optical breadboard, with aluminum composite panels to exclude ambient light from the sampling area, which includes the LED lights, camera, reflectance panel, and sample tray. The entire assembly measures approximately 56 × 45 × 60 cm and can be easily moved for imaging on-site or in the field.

To operate the system, a graphical user interface was written using LabVIEW’s Vision Development Module (VDM). An image and spectral plot are displayed as the instrument is scanning a sample. The system software also employs LabVIEW (v2022, National Instruments, Austin, TX, USA), running on Windows 11 (Microsoft Corporation, Redmond, Washington, U.S.A.). Linear stage movement, LED light control, and camera control were implemented with software development kits (SDKs) from hardware manufacturers. As the linear motorized stage moves the sample tray underneath it, the hyperspectral camera continuously collects line-scan reflectance signals. For any given sample, both hyperspectral reflectance and fluorescence images are saved in separate files using a standard format of band interleaved by line (BIL). However, this study only involved reflectance images.

### 2.2. Preprocessing

**Figure 2:**
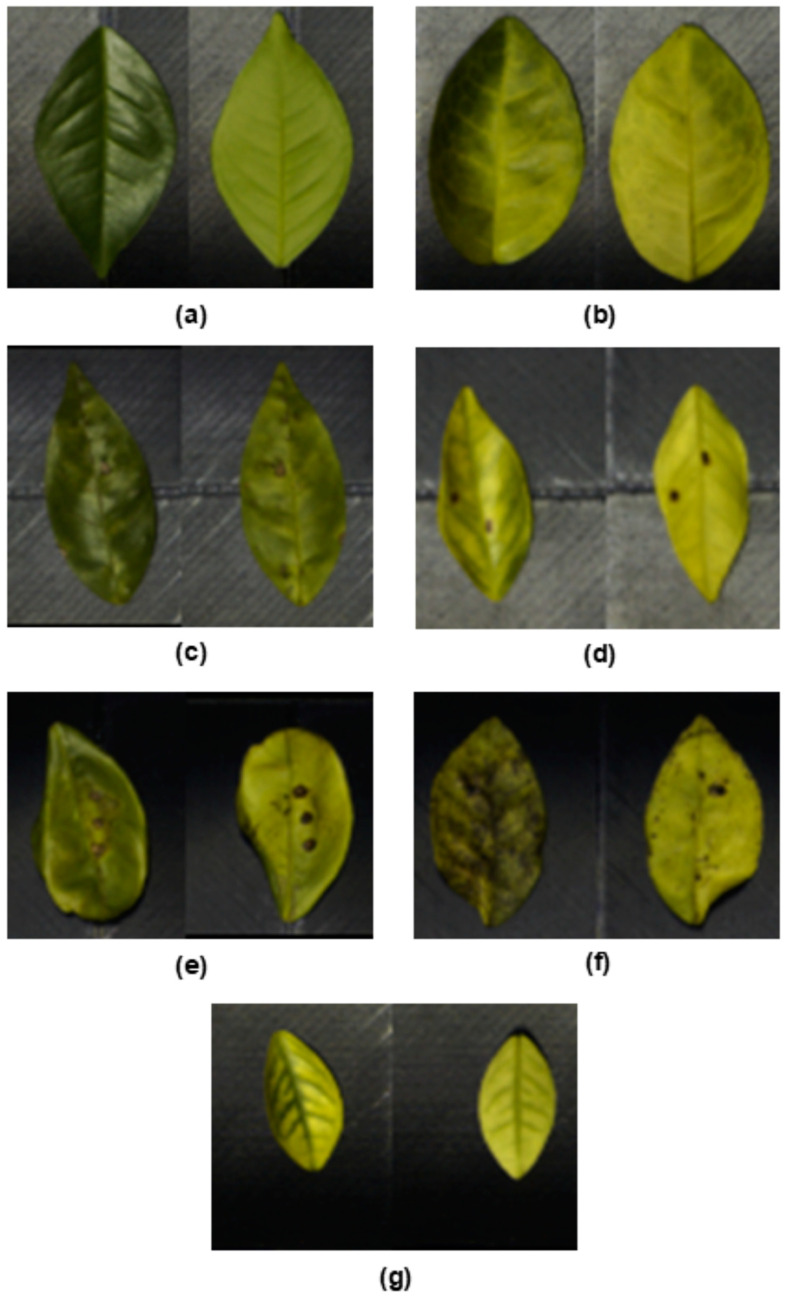
A pseudocolor sample leaf image from each class after preprocessing: (**a**) control/asymptomatic, (**b**) HLB, (**c**) scab, (**d**) greasy spot, (**e**) canker, (**f**) melanose, and (**g**) zinc deficiency.

Using a white calibration target affixed to the imaging tray. The raw reflectance values were converted to reflectance percentage. Also, the 348 spectral bands were binned, resulting in 116 spectral bands. The spatial dimension perpendicular to the linear motor axis was also binned to match the spatial resolution in that direction (1 mm per pixel). To facilitate dividing the images to leave a single leaf in each, a mask based on the saturation channel of a pseudocolor image was created. This also permitted the background pixel values to be set to 0. Next, the leading and trailing eight bands were removed from the dataset, since irregularities in the illumination spectrum had been observed. Likewise, images containing artifacts from camera overheating were discarded. The images of the front and back sides of each leaf in the dataset were then concatenated. Figure 2 displays a sample pseudocolor image from each class after preprocessing.

### 2.3. Band Selection

To test the effects of texture features, two ‘feature matrices’ were extracted for band selection. The first (the pixel matrix) of these was composed of all non-background image pixels, reshaped into a 2D matrix with rows corresponding to pixels and columns corresponding to bands. By implicitly considering each pixel a data point, any spatial information (i.e., the location of a pixel with respect to the rest of the leaf) and textural information was ignored.

The second feature matrix was formed by extracting five gray-level co-occurrence matrix (GLCM) texture features from each band and appending them as a row. The GLCM quantifies occurrences of pairs of pixel values adjacent in an image and is a well known means of extracting texture features from imagery. Several statistical features can be extracted from the GLCM, and five were selected for this study: contrast, dissimilarity, homogeneity, correlation, and angular second moment (ASM). Formulae for these features can be found in Gómez-Flores et al. [43]. Thus, each row of this second feature matrix (the GLCM matrix) corresponds to an HSI image, and each column to a feature from a specific band. Both feature matrices were standardized.

For band selection, analysis of variance (ANOVA) was employed to test whether the difference between the means of classes in each feature is significant using the F-statistic, thus quantifying the separability of the classes if that feature is retained. In this application, the features are wavelengths (using the first feature matrix) or texture features from wavelengths (using the second feature matrix). Typically, only features whose F-statistic exceeds a selected significance level would be selected and retained. However, two constraints specific to this study informed a novel selection method:

- Adjacent bands were expected to contain much redundant information;
- Exactly three bands were needed regardless of how many bands were significant (due to limitations of YOLOv8).

**Figure 3:**
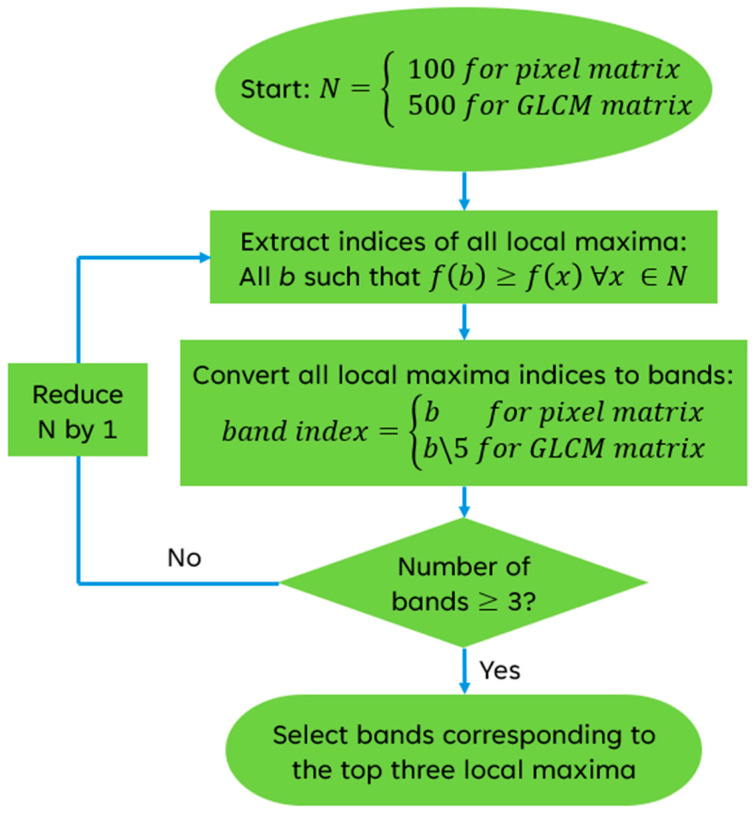
The algorithm designed for band selection. In this figure, f(x) refers to the F-statistic of a feature x, and N refers to the size of the local maximum neighborhood.

To address the first constraint, instead of the highest F-statistic, only local maxima of a neighborhood of F-statistics were selected, which will mitigate the possibility of selecting adjacent (and likely redundant) bands. Adjusting the size of the neighborhood will affect the number of local maxima selected, which is equivalent to the number of bands selected. A neighborhood size equivalent to the complete range will return a single value—the global maximum. To ensure that three bands would be selected, the neighborhood size was lowered until three local maxima were found. The indices of these maxima directly correspond to HSI bands. This selection criterion is summarized in Equation (1) as follows:

(1) $$\left\{b\in B|\forall x\in N,f\left(x\right)\leq f\left(b\right),\left|B\right|=3\right\}$$

where N is the neighborhood, f(x) is the F-statistic for a feature index x, B is the set of feature indices, and b is a feature index. Bands were selected from both feature matrices’ F-statistics with this method. For the first feature matrix, the chosen feature indices were equal to the band indices, but for the GLCM feature matrix, the integer quotient of the feature index with a divisor of five was the band index, since five features were extracted from each band. This process is displayed in Figure 3.

**Figure 4:**
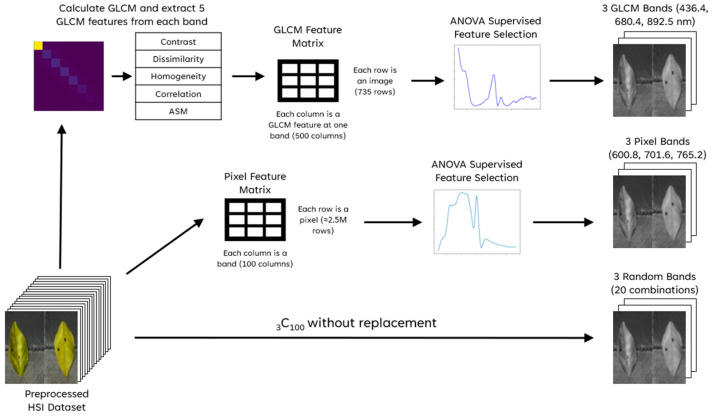
Summary of band selection methods.

The bands chosen using the GLCM feature matrix are referred to as GLCM bands, and those from the pixel intensity matrix as pixel bands. Also, to provide a comparison for the performance of each class, 20 random combinations of three bands were selected, and a model was trained on each. Figure 4 summarizes the band selection workflow.

### 2.4. Network and Training Parameters

The ‘nano’ and ‘small’ versions of the YOLOv8 architecture [60] were utilized since prior work suggests that these versions are better suited for embedded applications without degradation of performance. A model of each size was trained on each ANOVA band combination. This isolated the effects of model size from the effects of band selection. The dataset was randomly split according to a 70/15/15 ratio into training, validation, and test data. To eliminate the effect of the data split on comparisons of models and bands selected, the same split was employed to train and test all models in this study.

The models using the random band combinations used default training hyperparameter settings. For all other models, mutation-based tuning was employed to set hyperparameters. These were initialized to defaults with the following exceptions: Four parameters controlling augmentation—‘flipud’, ‘degrees’, ‘shear’, and ‘perspective’—were set to 0.5, 1.0, 0.1, and 0.1, respectively. ‘Mixup’ and ‘copy/paste’, being object detection parameters, were set to 0. All models were trained for a maximum of 300 epochs but permitted to stop early with a patience value of 50 epochs. Also, the previously extracted feature matrices were used only for band selection and were not involved in model training.

### 2.5. Performance Evaluation

For practical inspection purposes, type 1 errors are much less consequential than type 2 errors. Thus, recall and precision were recorded and analyzed for each class. Performance metrics for this study also included overall model accuracy and F1 score. The formulae for these, in terms of false positives (FP), true positives (TP), false negatives (FN), and true negatives (TN), are stated in Table 1.

## 3. Results

### 3.1. Band Selection

**Figure 5:**
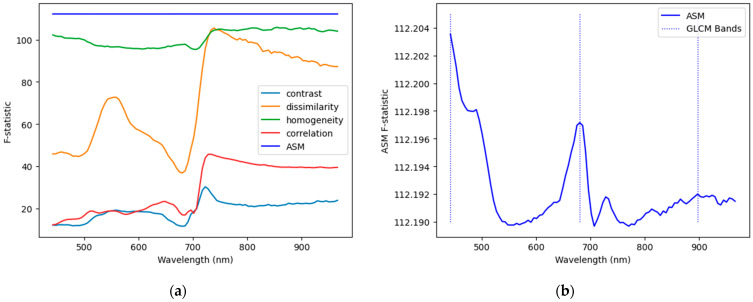
GLCM feature F-statistics and bands selected: (**a**) F-statistics for all GLCM features; (**b**) F-statistics for the ASM features only.

The ANOVA F-statistics of the ASM features were all considerably higher than those of any of the other four GLCM features. Since the scoring of bands was univariate, no adjustment was made to the band selection procedure, and the three bands corresponding to the local maxima of the F-statistic curve were selected. These wavelengths were 441.7, 680.4, and 897.8 nm and are shown with the F-statistics in Figure 5.

**Figure 6:**
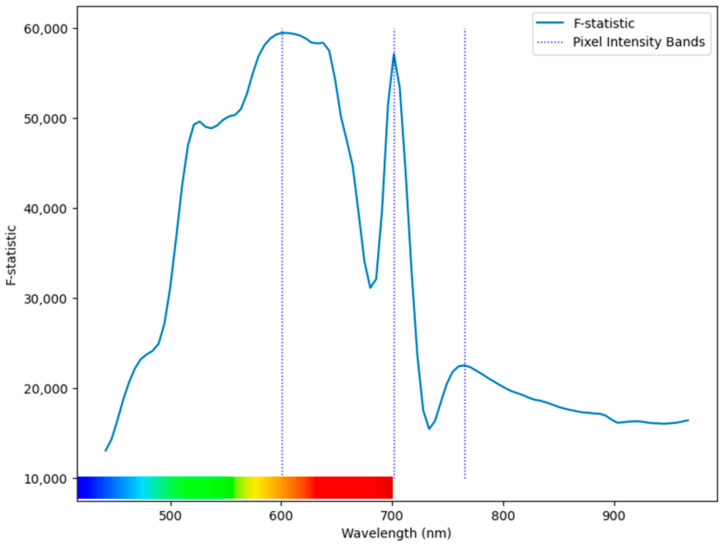
Pixel-based F-statistics and resulting bands.

The pixel feature band selection method yielded wavelengths of 600.8, 701.6, and 765.2 nm. The F-statistics for the pixel feature matrix and the resulting bands are plotted in Figure 6.

Previous band selection work on this dataset also selected the 441.7 and 600.8 nm bands but did not select any band above 722.8 nm. Supervised bands maximizing separability ranged from 489.5 to 600.8 nm [58]. This departure from the green range of the spectrum, especially for the pixel bands, is a result of using local extrema to counteract the selection of adjacent bands—the range of previously selected bands all have F-statistics greater than 45,000. Separability indicated by the peak at 765.2 nm was apparently not recognized by the previous methods.

### 3.2. Classification

**Figure 7:**
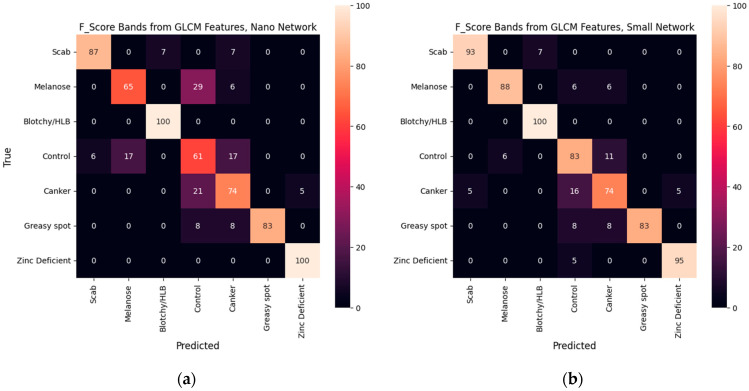
Confusion matrices of classification models using GLCM features reported in percent of true samples with (**a**) the nano network and (**b**) the small network.

**Figure 8:**
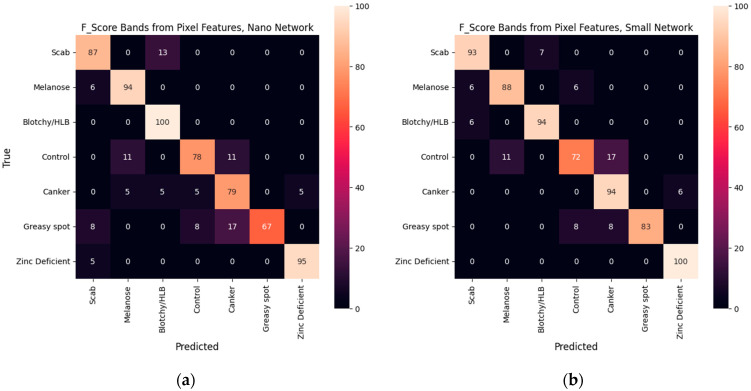
Confusion matrices of classification models using pixel (intensity) features reported in percent of true samples with (**a**) the nano network or (**b**) the small network.

**Figure 9:**
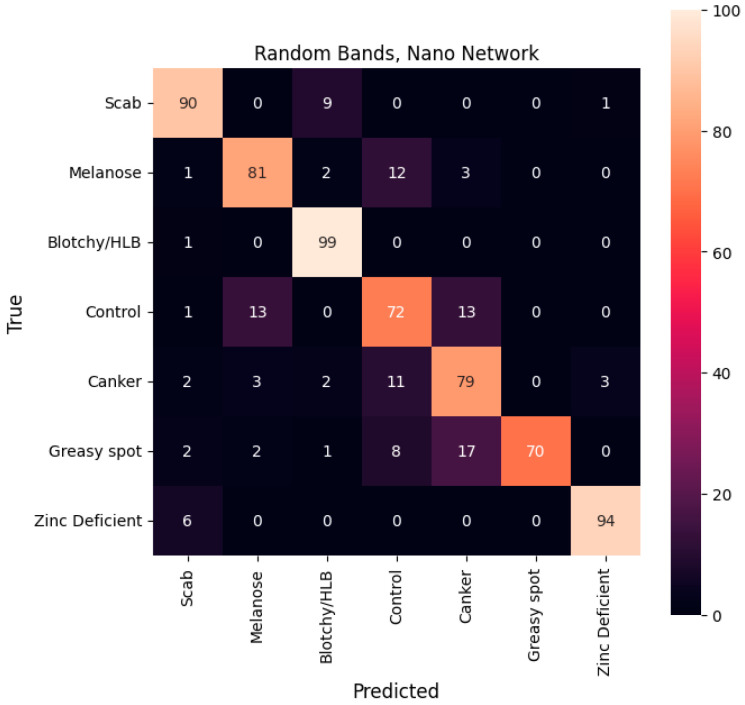
Mean confusion matrix of classification models using three random bands, reported in percent of true samples.

Table 2 summarizes the overall performance of each model, including the mean accuracy and weighted F1 score. Figure 7, Figure 8 and Figure 9 display the percentage of images from each test set class that were classified into each class for all five models.

**Figure 10:**
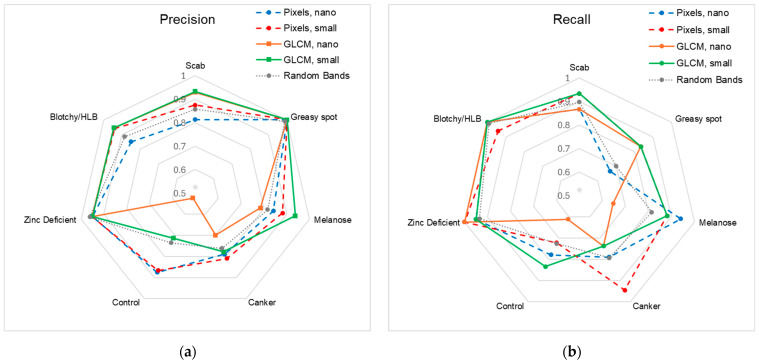
(**a**) Precision and (**b**) recall of all models. Results for the random bands are reported as means of all 20 models.

**Figure 11:**
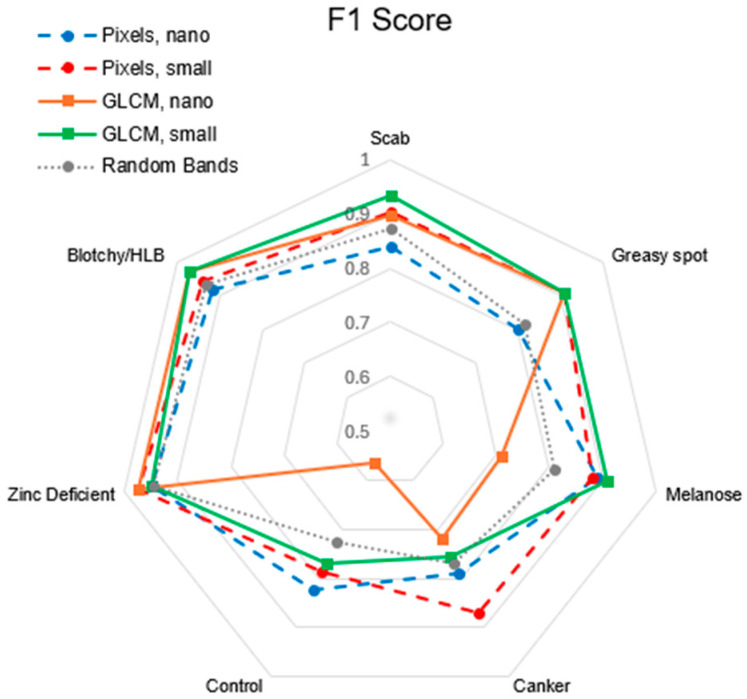
F1 Scores of all models. For the random band combinations, mean results are reported.

Figure 10 and Figure 11 display the class-specific results for each model. Distance from the center of the plots indicates higher values—for instance, for precision in greasy spot, the lines for each model lie at the edge of the plot. These metrics are also listed in Table 3, with results from random bands being means. Overall performance differences primarily stem from the control, canker, melanose, and greasy spot classes.

## 4. Discussion

Network size exerts a greater effect on classification performance than did the bands selected. Each of the small models exceeded an F1 score of 0.88, whereas performance with the nano architecture varied between 0.82 and 0.86. The greatest difference between the highest-performing model (pixel bands with a small network) and the others is observed in the recall of canker and greasy spot. Only for greasy spot did the GLCM bands show any clear advantage in recall. For small models, the GLCM bands displayed slightly improved recall in HLB, scab, and melanose, but considerably worse recall of canker. The nano model using GLCM bands was outperformed in control, canker, and melanose by both other nano models. Generally, it was expected that the larger model would perform better as long as training data permitted. However, the different band combinations likely accentuated the defects differently, resulting in varying potential for overfitting of the models.

Recall of HLB was nearly equal for all models, as was precision of greasy spot. This can probably be attributed to HLB covering the majority of the leaf surface, while greasy spot and melanose have very distinct discoloration regions, making them less likely to be hidden by the 1 mm/pixel resolution. Zinc-deficient leaves, despite displaying a similar color to HLB, are generally smaller and leave more pronounced and visible leaf veins compared to HLB (see Figure 2). As a result, this class was the best classified by all models.

For each model, the control class created a major error mode, being misclassified into melanose and canker. Since practically all the leaves in the dataset will contain at least some healthy leaf area, classes whose symptoms are small lesions are particularly likely to be confused with control. The resolution of the imagery probably exacerbated this occurrence. With the GLCM bands, canker was also often mistaken for control, but with the pixel bands, the canker leaves were not misclassified into melanose or control. The pixel bands likely permitted extraction of better features from the canker lesions, leading to less confusion among the canker class and other classes.

Even without the benefit of hyperparameter tuning, the random band combinations generally matched the performance of the nano model using pixel bands. Only for control and melanose did the nano model with pixel bands substantially outperform the mean random model. As can be seen in Figure 6, several bands surrounding 600 nm presented similar F-statistics. It is likely that these bands contain similar information and that combinations including any of them in place of 600.8 nm would yield similar performance. Similarly, compared to those of the other GLCM features, the range of the F-scores of the ASM features (<1) is very small relative to the values (≈112). This indicates that despite the classes being more separable with ASM than with any other GLCM feature, no band offers a substantial classification advantage over any other bands using ASM.

Since ASM was shown to yield the highest F-statistics, no other GLCM features influenced band selection. However, it is not apparent whether and/or to what extent ASM corresponds to the textural features the classifier networks learned to exploit. Bands favored by the other textural features may complement YOLOv8 better. On a similar note, most models accurately identified HLB and zinc deficiency but confused control, canker, and melanose. The band selection methods tested in this study treat all classes equally, despite two being the primary focus. Bands could instead be chosen to target specific classes or major classification error modes. Despite little overall effect on this dataset, the band selection procedure developed can easily be generalized to other feature extractors, feature scoring methods, numbers of input bands, or numbers of desired bands. It should be utilized for supervised band reduction in future studies. A limitation of the classification approach is that each leaf can only be assigned to one disease class. This is undesirable, since citrus diseases differ in frequency of occurrence and severity, and symptoms of multiple diseases can be found on the same leaf. Training a future model for semantic segmentation of leaves into diseased and healthy regions would permit detection of different infections on the same leaf and avoid the possibility of false negatives in more critical pathogens. Also, some disease classes (specifically melanose and greasy spot) and citrus black spot can present lesions approximately the size of (or even smaller than) the pixels in this dataset. Higher spatial resolution is needed to ensure detection of small lesions.

Although the mean results of the 20 random combinations of three bands were only slightly worse than those of the other models of the same network size, they do not rule out a subset of wavelengths permitting significantly better classification than the others. It is likely that the mean performance results from a mixture of ‘better’ and ‘worse’ band choices. Further study might seek to empirically identify this subset by testing regularly spaced bands to isolate the effect of certain regions of the spectrum.

## 5. Conclusions

Management of citrus pathogens requires distinguishing HLB and canker from other citrus leaf conditions. Early detection of HLB and canker symptoms informs more intelligent management by permitting earlier actions in response to infections and measurement of the effects of different management tactics. Also, the ability to phenotype these diseases can accelerate the development of varieties of citrus resistant to them.

Hyperspectral imaging combined with deep-learning-based analysis permits accurate classification of these infections. A novel supervised band selection method was developed and applied to select three wavelengths from hyperspectral imagery of both sides of citrus leaves, using both intensity and texture features. YOLOv8 was trained to classify these images, reaching an accuracy of 89.0% with a small YOLOv8 network and pixel intensity-based bands. With all band combinations, zinc deficiency and HLB were the best distinguished classes. Both ANOVA-based band combinations classified HLB with a recall and F1 score of at least 0.941 and 0.919, respectively. Confusion between canker and control lowered the texture-based band combination’s overall accuracies to 81.36% and 88.13% for the nano and small networks, respectively. Thus, these results fail to demonstrate that involving texture in the band selection process as opposed to intensity offers any performance improvement in HLB and canker detection. However, higher recall of greasy spot indicates that texture features are significant at the classification stage for mitigating confusion with other diseases. But for HLB and canker, the quality of texture features is likely uniform over the spectrum of this dataset and thus need not inform future band selection techniques. Choosing the larger network (‘small’) significantly improved performance compared to the ‘nano’ network in this case but brings the risk of overfitting on smaller datasets, as the authors have encountered in previous studies. Also, memory and processing requirements of larger networks may diminish suitability for in-field, embedded applications.

## Figures and Tables

**Table 1:** Metrics employed to gauge classifier network performance.

Metric	Accuracy	Precision	Recall	F1 Score
Expression in terms of TP, FP, TN, and FN	$\frac{TP+TN}{TP+FP+TN+FN}$	$\frac{TP}{TP+FP}$	$\frac{TP}{TP+FN}$	$\frac{2TP}{2TP+FP+FN}$

**Table 2:** Overall performance of all models. For the random band results, means and standard deviations (σ) are reported.

Band Selection Method	Wavelengths (nm)	Model Size	Accuracy (%)	Weighted F1 Score
GLCM Features	436.4, 680.4, 892.5	Nano	81.36	0.8156
		Small	88.13	0.8852
Intensity Features	600.8, 701.6, 765.2	Nano	86.44	0.8622
		Small	88.98	0.8959
Random Band Combinations		Nano	84.19 (σ = 4.3%)	0.8406 (σ = 0.044)

**Table 3:** Precision, recall, and F1 scores for each class and each model.

	Band Selection Method and Model Size	Class						
		Control	Scab	Greasy Spot	Melanose	Canker	Zinc Deficient	HLB
Precision	Pixels, nano	0.875	0.813	1.000	0.842	0.789	0.950	0.850
	GLCM, nano	0.524	0.929	1.000	0.786	0.700	0.952	0.944
	GLCM, small	0.714	0.933	1.000	0.938	0.778	0.950	0.944
	Pixels, small	0.867	0.875	1.000	0.882	0.810	0.952	0.941
	Random bands	0.736	0.857	0.989	0.816	0.762	0.961	0.886
Recall	Pixels, nano	0.778	0.867	0.667	0.941	0.789	0.950	1.000
	GLCM, nano	0.611	0.867	0.833	0.647	0.737	1.000	1.000
	GLCM, small	0.833	0.933	0.833	0.882	0.737	0.950	1.000
	Pixels, small	0.722	0.933	0.833	0.882	0.944	1.000	0.941
	Random bands	0.725	0.897	0.700	0.815	0.792	0.935	0.991
F1 Score	Pixels, nano	0.839	0.800	0.889	0.789	0.824	0.950	0.919
	GLCM, nano	0.897	0.909	0.710	0.718	0.564	0.976	0.971
	GLCM, small	0.933	0.909	0.909	0.757	0.769	0.950	0.971
	Pixels, small	0.903	0.909	0.882	0.872	0.788	0.976	0.941
	Random bands	0.873	0.816	0.811	0.771	0.726	0.947	0.934

## Data Availability

The datasets presented in this article are not readily available because they are part of ongoing research, as well as due to obligations of the funding agreement. Requests to access the datasets should be directed to Thomas Burks.
